# Lippia javanica (Burm. F.) Herbal Tea: Modulation of Hepatoprotective Effects in Chang Liver Cells via Mitigation of Redox Imbalance and Modulation of Perturbed Metabolic Activities

**DOI:** 10.3389/fphar.2023.1221769

**Published:** 2023-08-07

**Authors:** Veronica F. Salau, Ochuko L. Erukainure, Kolawole A. Olofinsan, Recardia L. S. Schoeman, Motlalepula G. Matsabisa

**Affiliations:** ^1^ Department of Pharmacology, University of the Free State, Bloemfontein, South Africa; ^2^ Department of Biochemistry, University of KwaZulu-Natal, Durban, South Africa; ^3^ Laser Research Centre, Faculty of Health Sciences, University of Johannesburg, Doornfontein, South Africa

**Keywords:** oxidative stress, hepatotoxicity, gluconeogenesis, antioxidants, cholinergic enzyme

## Abstract

**Introduction:** Hepatic oxidative injury is one of the pathological mechanisms that significantly contributes to the development of several liver diseases. In the present study, the hepatoprotective effect of *Lippia javanica* herbal tea was investigated in Fe^2+^- mediated hepatic oxidative injury.

**Methods:** Using an *in vitro* experimental approach, hepatic oxidative injury was induced by co-incubating 7 mM FeSO^4^ with Chang liver cells that have been pre-incubated with or without different concentrations (15–240 μg/mL) of *L. javanica* infusion. Gallic acid and ascorbic acid served as the standard antioxidants.

**Results:** The infusion displayed a reducing antioxidant activity in ferric-reducing antioxidant power (FRAP) assay and a potent scavenging activity on 2,2-diphenyl-2- picrylhydrazyl (DPPH) radical. Pretreatment with *L. javanica* infusion significantly elevated the levels of reduced glutathione and non-protein thiol, and the activities of superoxide dismutase (SOD) and catalase, with concomitant decrease in hepatic malondialdehyde levels, acetylcholinesterase, glucose-6-phosphatase, fructose-1,6-bisphosphatase, glycogen phosphorylase and lipase activities. The infusion showed the presence of phytoconstituents such as phenolic compounds, tannins, phenolic glycosides and terpenoids when subjected to liquid chromatography—mass spectrometry analysis. Molecular docking revealed a strong binding affinity of dihydroroseoside and obacunone with both SOD and catalase compared to other phytoconstituents.

**Conclusion:** These results portray a potent antioxidant and hepatoprotective effect of *L. javanica*, which may support the local usage of the herbal tea as a prospective therapeutic agent for oxidative stress-related liver diseases.

## Introduction

About two million cases of global mortality are attributed to liver diseases, with liver cirrhosis and liver cancer being the most common causes of these deaths (Asrani et al., 2019). Besides increased risks of mortality, chronic liver diseases cause several extrahepatic morbidities which contribute notably to low quality of life. Thus, liver diseases, though underestimated, pose a high economic burden which is a major concern (Stepanova et al., 2017; Asrani et al., 2019).

Regardless of the cause, most chronic liver diseases are typified by oxidative stress (Cichoż-Lach and Michalak, 2014). Excessive reactive oxygen species (ROS) cause disturbances in redox homeostasis which results in oxidative stress, a major pathological mechanism involved in the development and progression of several liver diseases. Oxidative stress induces dire alterations in liver proteins, lipids and DNA components as well as impair pathways involved in normal biological functions of the liver (Li et al., 2014; Li et al., 2015). The liver is the main organ usually attacked by ROS, as the parenchymal cells, hepatic stellate cells, Kupffer cells and endothelial cells of the liver are all vulnerable to oxidative injury, causing damages to each cell types (Cichoż-Lach and Michalak, 2014). Several risk factors including drugs, alcohol, irradiation and environmental pollutants such as heavy metals may mediate hepatic oxidative stress. Damages induced by oxidative stress significantly contribute to impairment of gene expression and progression of liver diseases as well as apoptosis and necrosis (Cichoż-Lach and Michalak, 2014; Li et al., 2015).

Severe disturbances in hepatic glucose and lipid metabolism homeostasis have been recognized as some of the major mechanisms involved in liver diseases such as liver cirrhosis, liver steatosis and fatty liver, with oxidative stress being a key contributor (Miksztowicz et al., 2012; Ding et al., 2018). Excess cellular levels of glucose and lipids can serve as substrates for the generation of glucotoxic and lipotoxic species, respectively, which can cause damage to biomolecules, induce metabolic stress and eventual cell death (Mota et al., 2016; Chen et al., 2020). Additionally, altered cholinergic enzyme activities have been implicated in the pathogenesis of liver diseases and studies have reported oxidative stress as facilitator of cholinergic dysfunction (Garcia-Ayllon et al., 2012; Erukainure et al., 2021a). These corroborates the use of antioxidants as therapies for targeting oxidative stress in the management of liver diseases (Upadhyay et al., 2022).

Several medicinal plants, including herbal infusions have been globally used over the decades for the treatment of several chronic liver diseases due to their availability, curative effects and minute adverse effects. These therapeutic characteristics have been ascribed to the phytochemical components of the plants (Hong et al., 2015; Chukwuma et al., 2019). Studies have indicated that medicinal plants and their phytochemicals exhibit their hepatoprotective effects in several ways including mitigation of oxidative stress, blockage of fibrogenesis and suppression of tumorigenesis (Dhiman et al., 2012; Hong et al., 2015).

*Lippia javanica* (Burm.f.) (Family: Verbenaceae) is a multi-stemmed, woody, drought-resistant shrub that is naturally distributed in central, eastern and southern Africa including South Africa, Malawi, Botswana, Kenya, Zambia, Angola, Zanzibar, Tanzania and Mozambique, as well as tropical Indian sub-continent (Germishuizen et al., 2006; Shahriar et al., 2014). In South Africa, it is widely distributed in different provinces which include KwaZulu-Natal, Gauteng, Free State, Limpopo, Eastern Cape and Northwest. It is known as one of the aromatic indigenous shrubs in South Africa. The common names of *L. javanica* include fever tree, wild sage, wild tea and lemon bush. The Xhosa community of South Africa call it *inzinzinba* while the Zulus call it *umwazi* (Maroyi, 2017). Traditionally, *L. javanica* has been used from time immemorial as herbal tea or as either root or leave decoction to treat fever, malaria, cough, cold, chest pain, asthma, bronchitis, and diarrhea. The Zulus in South Africa use the herbal tonic as an immune booster. In Zimbabwe and South Africa, the burnt whole plant or leaves are used as mosquito repellant (Lukwa et al., 2009; Maroyi, 2017). The reported pharmacological activities of the plant include antioxidant, antimalarial, antidiabetic, anticancer, antiviral and antimicrobial activities (Fouché et al., 2008; Mujovo et al., 2008; Shikanga et al., 2010; Maroyi, 2017). The neuroprotective effect of its herbal tea infusion on lead-induced brain oxidative damage in Wistar rats was also reported by Suleman et al. (2022). Despite its numerous documented medicinal properties, there is limited information on the effect of *L. javanica* on liver diseases.

The purpose of the present study was to investigate the potential hepatoprotective effect of *L. javanica* tea infusion on iron-induced oxidative hepatic injury in Chang liver cells by assessing its effect on oxidative stress, cholinergic dysfunction, altered carbohydrate metabolism and lipase activities.

## Materials and methods

### Plant material

#### Plant collection and verification

*Lippia javanica* (Burm.f.) Spreng leaves were collected from Langenhoven Park, Bloemfontein, Free State Province, South Africa (GPS Coordinates: 29°05′32.2″S 26°09′25.6″E) by Prof. M.G. Matsabisa. The plant sample was deposited at the Geo Potts Herbarium, University of the Free State, Bloemfontein 9,300, South, where it was identified, authenticated and assigned a voucher specimen number (BLFU/MGM005).

#### Plant infusion preparation

After air-drying at room temperature, the *L. javanica* leaves were pulverized into powder. Extraction was done by boiling 50 g of the leave powder in 500 mL of distilled water for 10 min. The mixture was allowed to cool and then filtered into a pre-weighed glass beaker using a Whatman filter (Whatman, England). The extract was concentrated in a water bath at 50°C. The dry plant infusion was scrapped and transferred into a glass vial and stored at −20°C.

The infusion was re-constituted in distilled water by preparing a stock solution of 1 mg/mL from which various working concentrations ranging from 15 to 240 μg/mL were prepared for different assays. Similarly, the same working concentrations were prepared for two antioxidant standards, ascorbic acid and gallic acid from a stock solution of 1 mg/mL.

### Phytochemical characterization and quantification

#### Total phenolic content

The total phenolic content of the infusion was determined using the Folin-Ciocalteu’s phenol reagent as described by McDonald et al. (2001). In brief, 40 µL of 240 μg/mL plant extract was incubated in the dark with 200 µL of 10% Folin Ciocalteau reagent and 160 µL of 0.7 M Na_2_CO_3_ for 30 min at room temperature. The absorbance of the triplicates were measured at 765 nm, using a Multiskan ascent plate reader (Thermo scientific, S.A). The total phenolic content was estimated from a gallic acid standard curve and results were expressed as gallic acid equivalents (GAE) in milligrams per gram of dry weight.

#### Total flavonoid content

The total flavonoid content of the infusion was estimated by utilizing the aluminum chloride colorimetric method described by Chang et al. (2002), with slight modification. Briefly, 100 μL of the infusion (240 μg/mL) was added to a mixture of 100 μL of methanol, 10 μL of aluminum chloride, 10 μL of 1 mol/L potassium chloride and 200 μL of distilled water. The mixture was allowed to stand for 30 min at room temperature. The absorbance of the independent triplicates were measured at 415 nm and the total flavonoid content was estimated from a quercetin calibration standard curve. The results were expresses as quercetin equivalent (QE) in milligrams per gram of dry weight.

#### Liquid chromatography-mass spectrometry (LC-MS) analysis of *L. javanica*

The pharmacologically active chemical constituents present in the L. *javanica* infusion were characterized via direct-loop injection into Shimadzu LC/MS-2020 Single Quadrupole Liquid Chromatograph Mass Spectrometer (LCMS) equipped with an electrospray ionization (ESI) source. The analysis data acquisition duration was set at 50 min at a low-pressure gradient, while the LC photodiode array (PDA) sampling frequency was kept at 1.5625 Hz. The oven temperature range was maintained between 40°C–50°C, and the pump flow rate was kept at 300 μL/min. The mobile phase solvent system contained 0.1% formic acid in water (phase A) and methanol: acetonitrile (1:1) (phase B). Scanning was done at positive and negative polarities with other operating parameters which include, Start Time: 0.0 min; End Time: 50.0 min; Event Time: 0.25 s; Cell Temperature: 40°C; Threshold: 0; Start Wavelength: 220 nm; End Wavelength: 400 nm; Detector Voltage: +0.00 kV; Scan Speed: 5,000 u/s; Start and End m/z: 100.0 and 1,000.0, respectively. The compounds were identified by direct comparison of the generated spectra containing the relative abundance and the m/z fragmentation patterns with those in the m/z cloud database at https://www.mzcloud.org/.

### *In vitro* antioxidant activities of the infusion

#### 2,2-Diphenyl-1-picrylhydrazyl (DPPH) scavenging activity

The free radical (DPPH) scavenging activity of the infusion was determined by using a previously established protocol (Changlian et al., 2000). In brief, 50 μL of 0.3 mM DPPH in methanol was mixed with 100 μL of different concentrations (15–240 μg/mL) of the infusions or the standards ascorbic acid and gallic acid in a 96-well plate. The plate with the samples was incubated in the dark at room temperature for 30 min. The absorbance was measured against a blank solution at 517 nm, and the percentage scavenging activity was calculated.

#### Ferric reducing antioxidant power (FRAP)

The ferric-reducing capacity of the infusion was determined by the potassium ferricyanide method according to Benzie and Strain (1996) with slight modifications. 20 μL of different concentrations (15–240 μg/mL) of the herbal infusion was incubated with 20 µL of 0.2 M sodium phosphate buffer (pH 6.6) and 20 µL of 1% potassium ferricyanide at 50°C for 30 min 10% trichloroacetic acid (20 µL) was then added to the reaction mixture to acidify it. An aliquot of the acidified sample was added to 20 µL of distilled water and 20 µL of 10% FeCl_3_. The absorbance was read at 700 nm. The results were expressed as a percentage of the absorbance of the sample to the absorbance of gallic acid.

#### Cell lines

Chang liver cells (ATCC^®^ CCL-13™) were procured from the American Type Culture Collection (ATCC^®^), Manassas, Virginia, United States.

#### Cytotoxicity screening

Chang liver cells were seeded in a 96-well plate (Nunc, Thermofischer Scientific) at a cell density of 10,000 cells/well (100 µL/well) and left to attach overnight at 37°C in humidified atmosphere with a 5% CO_2_ concentration. The cells were then treated with various concentrations of *L. javanica* (15–240 μg/mL) and incubated for 48 h at 37°C. Doxorubicin (3 μg/mL) was used as a positive control. Dimenthylsulfoxide (DMSO) (0.5%) was used as a vehicle control. The cytotoxicity of the cells were evaluated using MTT [3-(4,5-dimethylthiazol-2-yl)-2,5-diphenyltetrazolium bromide. After treatment, the spent media was aspirated and replaced with 0.5 mg/mL MTT solution (100 µL/well) dissolved in fresh media followed by an incubation at 37°C for 2 h. After the incubation, the MTT solution was removed and 100 µL of dimenthylsulfoxide was added. Subsequently, the absorbance was read at 550 nm using the Multiskan GO spectrophotometer (Thermo Scientific). The results were expressed as mean ± SD the percentage cell viability of biological repeats.

#### Induction of oxidative stress in Chang liver cells

Oxidative stress was induced in Chang liver cells using a modified method from a previous protocol (Chukwuma et al., 2019). Briefly, the cells were seeded into 6-well plates (TPP^®^, Merck) at 160,000 cells/well (2 mL/well) and left to attach overnight at 37°C. After attachment, the cells were incubated with various concentrations of *L. javanica* extract or the standards, ascorbic acid and gallic acid (15–240 μg/mL) at 37°C for 25 min. Thereafter, 600 µL of 7 mM iron sulphate (FeSO_4_) was added to each well and the cells further incubated for 30 min at 37°C. Positive controls and negative controls were included and comprised of untreated cells with and without the addition of FeSO_4_. After incubation, the treatment was removed, and the cells washed twice with PBS (1 mL/well). The PBS was aspirated and the cells were dissociated using 200 µL of trypsin/EDTA and incubated at 37°C until the cells were completely dissociated. The trypsin was neutralized using 700 µL of complete media (DMEM with 10% FBS). The cell suspension was collected in 1.5 mL microcentrifuge tubes respectively and centrifuged at 17,000 × *g* for 12 min using the Hettich ^®^ MIKRO 120 centrifuge. The supernatant collected and stored at −80°C until further use.

#### Determination of oxidative stress biomarkers

Oxidative stress biomarkers were determined in the cell by analyzing the level of reduced glutathione (GSH), activities of superoxide dismutase (SOD) and catalase, as well as and malondialdehyde (MDA) level.

##### Reduced glutathione (GSH) level

GSH levels were determined in the hepatic cells by utilizing Ellman’s spectrophotometric method (Ellman, 1959) with slight modification. Briefly, 0.2 mL of the cell’s supernatant was mixed with 0.6 mL of trichloroacetic acid (10%) and centrifuged for 5 min at 1,000 × *g*. 0.2 mL aliquot of the supernatant (deproteinized solution) and 0.05 mL of Ellman’s reagent were placed in a 96 well microplate and incubated for 10 min at room temperature. Absorbance was read at 415 nm, and GSH protein level was estimated from a glutathione standard curve.

##### Superoxide dismutase (SOD) activity

The SOD activity in the cells were determined by utilizing a modified procedure of Kakkar et al. (1984). Briefly, a 96- well microplate containing the supernatant (15 μL), 100 µM diethylenetriaminepentaacetic acid (DETAPAC; 170 μL) and 15 μL of 1.6 mM hydroxydopamine (6-HD) was gently swirled before the absorbance was immediately measured at 492 nm thrice at 1 min interval.

##### Catalase activity

The hepatic catalase activity was measured in the supernatant by adopting the method of Hadwan and Abed (2016). Briefly, 100 μL of the supernatant was added to 1,000 μL of 0.065 μM H_2_O_2_ and incubated for 2 min at 37°C. 100 μL of 32.4 mM ammonium molybdate was used to terminate the reaction and the absorbance was read at 347 nm against the blank containing only H_2_O_2_.

##### Malondialdehyde (MDA) levels

Lipid peroxidation levels was determined by analyzing for MDA concentration according to previous method (Chowdhury and Soulsby, 2002). Briefly, 100 μL of the cell’s supernatant was added to a mixture containing 100 μL of 8.1% sodium dodecyl sulfate solution, 375 μL of 20% pure acetic acid and 1,000 μL of 0.25% thiobarbituric acid. The reaction mixture boiled for 1 h in water bath, and the absorbance was read at 532 nm after cooling, to estimate MDA levels.

#### Determination of non-protein thiol (NPSH) content

Hepatic non-protein thiol level was estimated spectrophotometrically using Ellman’s method (Ellman, 1959). Briefly, a mixture of 300 μL of cell supernatant and the same volume of 10% trichloroacetic acid were centrifuged at 1,000 × *g* for 5 min. 50 μL of the resulting supernatant, 150 μL of Ellman’s reagent and 50 μL of 0.1 M phosphate buffer were mixed and incubated for 10 min at 37°C. The absorbance was read at 412 nm and non-protein thiol content were calculate from standard curve of cysteine.

#### Determination of nitric oxide (NO) level

Hepatic nitric oxide level was determined based on Greiss method according to Tsikas (2005). Briefly a solution made up of 100 μL of the cell supernatant and equal volume of Greiss reagent in a 96 well-plate was incubated in the dark for 30 min at room at 25°C, using the same volume of distilled water as the blank. After incubation, absorbance of the solution was measured at 548 nm.

#### Determination of acetylcholinesterase activity

Acetylcholinesterase activity was determined in the hepatic cells by employing a previously established method (Ellman et al., 1961). Briefly, a solution containing 100 μL of the supernatant was added to 50 μL of 3.3 mM Ellman’s reagent (pH 7.0) and 250 μL of 0.1 M phosphate buffer (pH 8) was incubated for 20 min at 25°C. 50 μL of 0.05 M acetylcholine iodide was then added. The absorbance was immediately measured at 412 nm at 3 min intervals.

#### Determination of glucogenic enzymes activities

##### Glucose 6-phosphatase activity

Glucose 6-phosphatase activity was estimated in the hepatic cells according to a modified method of Balogun and Ashafa (2017). Briefly, 200 μL of the cell supernatant, 100 μL of 0.1 M glucose 6-phosphate, 200 μL of 5 mM KCl, and 1.3 mL of 0.1 M Tris-HCl buffer mixture was incubated at 37°C for 20 min. An addition of 1,000 μL 10% trichloroacetic acid was used to terminate the reaction, after which it was allowed to stand on ice for 10 min before centrifuging for 10 min at 5,000 g. 250 μL aliquot of the supernatant was placed in a 96-well plate and the absorbance was read at 340 nm.

##### Fructose-1-6-bisphosphatase activity

Fructose-1-6-bisphosphatase activity of the cells were determined according to a modified method by Balogun and Ashafa (2017). Briefly, 100 μL of the cell supernatant was transferred to tube containing 0.05 M 100 μL of fructose (0.05 M), 100 μL of potassium chloride (0.1 M), 250 μL of magnesium chloride (0.1 M), 1.2 mL of Tris–HCl buffer (0.1 M, pH 7.0) and 250 μL of Ethylenediaminetetraacetic acid (1 mM) and incubated for 15 min at 37°C. 10% trichloroacetic acid was used to halt the reaction and further centrifuged for 10 min at 5,000 g (4°C). Thereafter, 50 μL of 1.25% ammonium molybdate and 9% ascorbic acid was included in the reaction, it was allowed to stand for 20 min at room temperature. The absorbance was read at 680 nm.

##### Glycogen phosphorylase activity

The glycogen phosphorylase activity of cells were evaluated based on the procedure of Balogun and Ashafa (2017). Briefly, 200 µL of the cell supernatant, 100 µL solution of 64 mM glucose-1-phosphate and 100 µL 4% glycogen were mixed and incubated for 10 min at 30°C. 20% ammonium molybdate in concentrated sulfuric acid was used to terminate the reaction. Thereafter, Elon reducer and distilled water was added to the mixture and further incubated for 45 min at 30°C. The absorbance was measured at 340 nm.

#### Determination of lipase activity

Hepatic lipase activity in the cells was estimated according to a previous method with slight modifications (Kim et al., 2010). Briefly, 200 µL of the cell supernatant was added 390 µL of Tris buffer (pH 7.0) and incubated at 37°C for 15 min 100 μL of p-nitrophenyl butyrate in dimethylformamide (p-NPB) was then added to the mixture before incubating for another 15 min. The absorbance was measured at 405 nm at 1 min interval. Lipase activity of the hepatic cells was expressed as the rate of reaction (ΔA/min).

#### Molecular docking

This computer analysis was employed to determine the binding affinities of the phytochemicals of L. *javanica* infusion with catalase and SOD antioxidant enzymes. The x-ray diffraction structure of the respective proteins (1F4J and 2C9V) with 2.40 Å and 1.07 Å resolutions were retrieved from the Protein Data Bank (https://www.rcsb.org/). Non-proteins and water molecules co-crystalized with the proteins were removed using the Dock prep tool algorithm of the Chimera software (V.1.16). Next, the automated program also added hydrogen atoms and gasteiger charges, as described according to Wang et al. (2006). Subsequently, the 3D structure of the LC-MS identified chemical compounds in the L. *javanica* infusion were similarly downloaded from the Zinc database (https://zinc15.docking.org/substances/home/) and prepared using the previous software employed for the antioxidant enzymes. The catalytic pocket of the proteins were determined using the algorithm of the CASTp online server (http://cast.engr.uic.edu/) before molecular docking was carried out within a search volume covering X, Y, and Z dimension of 13 × 13 × 13 for SOD and 18 × 16 × 14 for catalase. Then, the calculated binding energies of the most stable ligand-protein complexes were recorded, and the 2D images of the various interactions involved were visualized using with BIOVIA Discovery Studio application.

#### Statistical analysis

The experiments were carried out in triplicate (*n* = 3) and results were presented as mean ± SD. Analysis of data was achieved by utilizing SPSS (Windows V25) and statistically significant difference between test groups was established at *p* < 0.05 using a one-way analysis of variance (ANOVA), followed by the use of Dunnett and Tukey’s HSD multiple range Post-hoc tests for comparison of experimental mean values.

## Results

The infusion extract of *L. javanica* revealed a total phenolic content of 25.95 ± 0.74 mg GAE/g and a total flavonoid content of 73.57 ± 2.12 mg QE/g of dry weight of extract. The extract showed a higher amount of flavonoid than phenolics.

**FIGURE 1:**
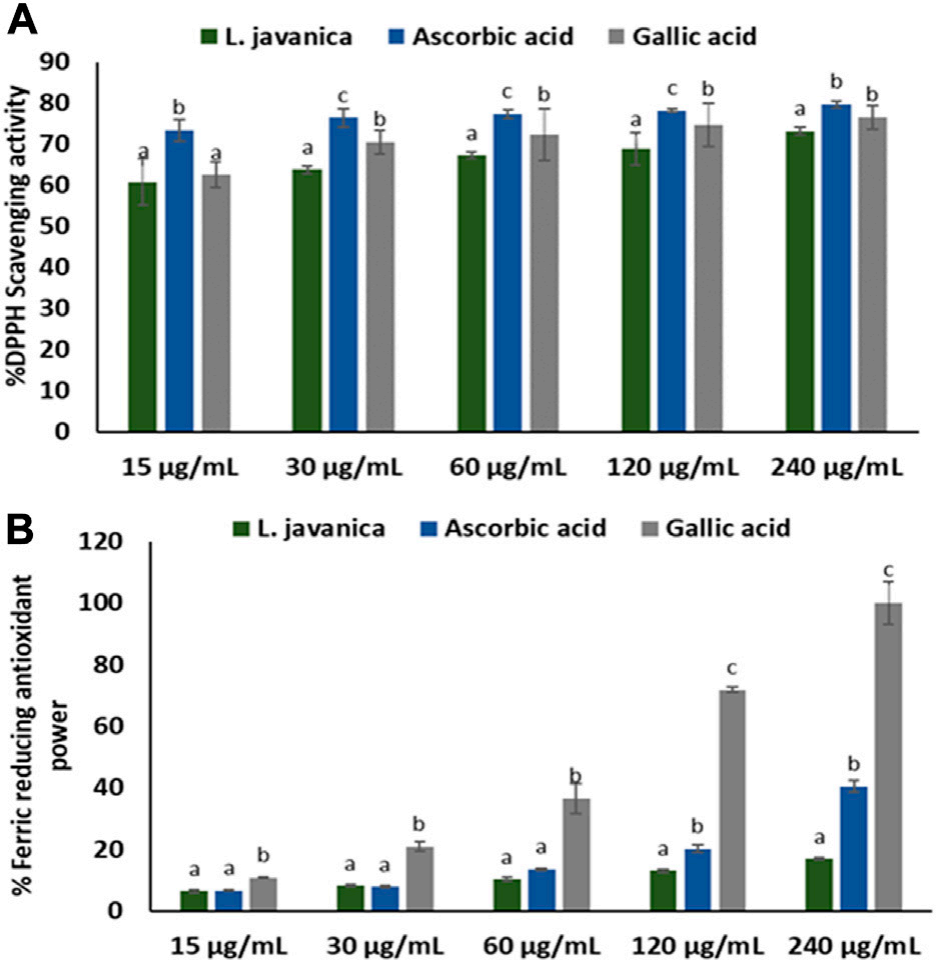
**(A)** DPPH scavenging and **(B)** FRAP activities of *L. javanica*. Data presented as mean ± standard deviation. Different unique alphabetical letters (a-c) above the bars for a given concentration illustrate the statistical significance of difference (*p* < 0.05), Tukey’s-HSD multiple range *post hoc* test).

As indicated in Figure 1A, *L. javanica* infusion significantly (*p* < 0.05) scavenged DPPH free radical at increasing concentrations, with a low IC_50_ value of 1.22 μg/mL (Table 1) and compared favorably with the standards, ascorbic acid (IC_50_: values of 0.11 μg/mL) and gallic acid (IC_50_: values of 0.61 μg/mL). However, the infusion only exhibited slight increase in Fe^3+^ reducing activity (Figure 1B) with IC_50_ value of ˃1,000 μg/mL) as compared to ascorbic acid at 120 and 240 μg/mL doses (IC_50_: values of 975.73 μg/mL) and gallic acid at all tested concentrations (IC_50_: values of 63.81 μg/mL).

**TABLE 1:** IC_50_ values of DPPH and FRAP activities of *L. javanica* herbal leaves.

Activities	*L. javanica*	Ascorbic acid	Gallic acid
DPPH	1.22	0.01	0.61
FRAP	>1,000	975.73	63.81

Values are expressed as µg/ml. DPPH: 2,2-diphenyl-1-picrylhydrazyl, FRAP: ferric reducing antioxidant power.

**FIGURE 2:**
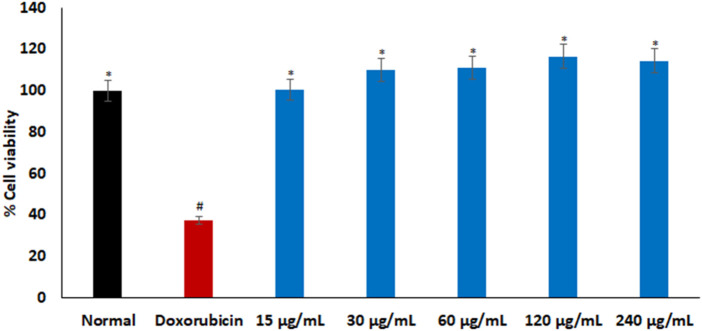
Cytotoxic effect of *L. javanica* on Chang liver cells. Value = mean ± SD; *n* = 3. *Statistically significant compared to doxorubicin group; #statistically significant compared to the normal control cell (*p* < 0.05, Dunnett’s multiple range *post hoc* test).

As portrayed in Figure 2, there was no significant difference in % cell viability between the cells treated with *L. Javanica* infusion and the normal Chang liver cells. Treatment with doxorubicin significantly (*p* < 0.05) reduced the cell viability when compared to the normal control.

**FIGURE 3:**
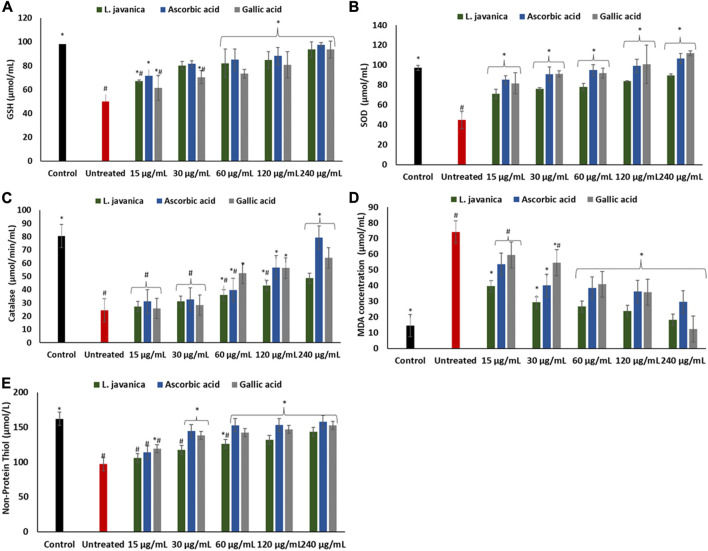
Effect of *L. javanica* on **(A)** GSH level **(B)** SOD activity, **(C)** catalase activity, **(D)** MDA level, and **(E)** non-protein thiol level in oxidative hepatic injury. Value = mean ± SD; *n* = 3. *Statistically significant compared to untreated hepatic cells; #statistically significant compared to the control cells (*p* < 0.05, Dunnett’s range *post hoc* test). GSH = Reduced glutathione, SOD = superoxide dismutase, MDA = Malondialdehyde.

As depicted in Figures 3A–E, the induction of oxidative injury in Chang liver cells using ferrous sulphate led to significant (*p* < 0.05) depletion in the levels of GSH and NPSH, activities of SOD and catalase, with a concurrent increase in MDA level when compared with the normal control. Pretreatment of the cells with *L. javanica* significantly (*p* < 0.05) reversed these levels and activities and compared favorably with the two standard antioxidants, ascorbic acid and gallic acid. However, there was no significant difference in the catalase activity and NPSH level of the untreated cells and *L. javanica*-treated cells at lower concentrations.

**FIGURE 4:**
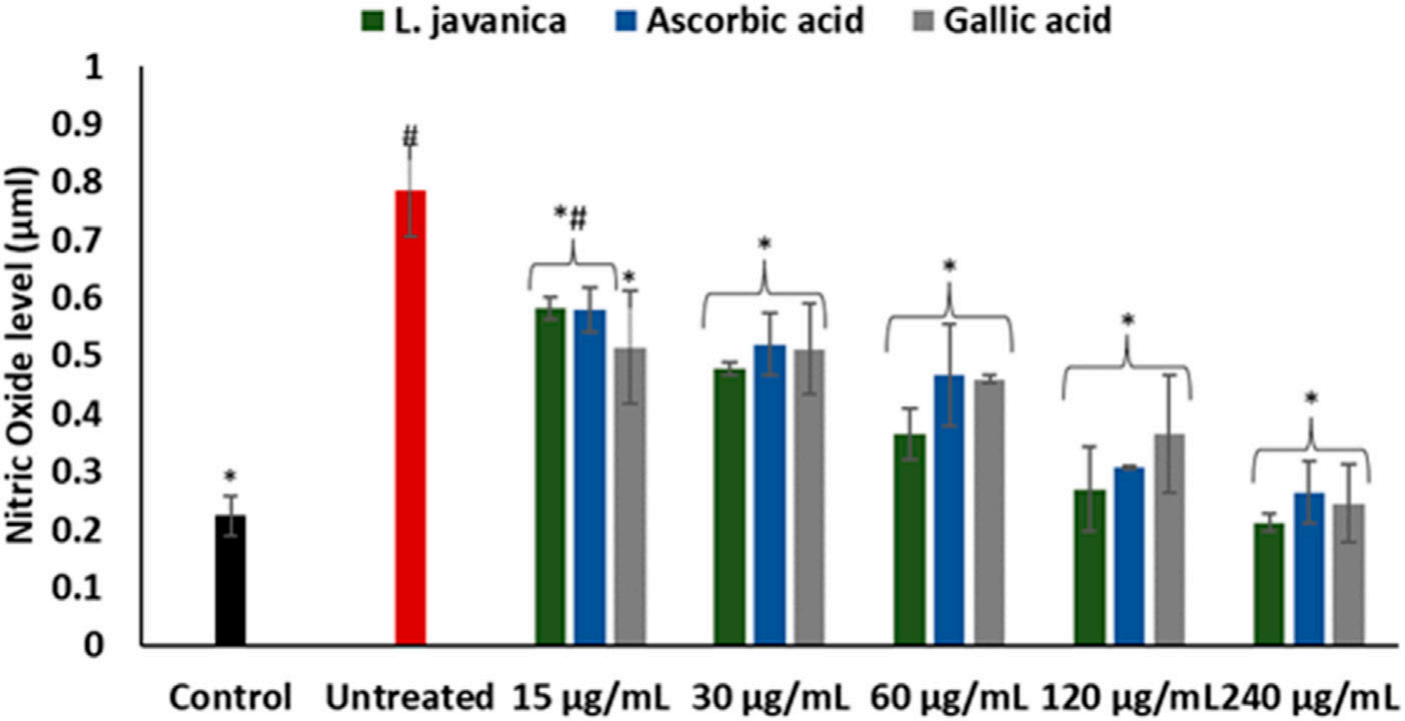
Effect of *L. javanica* on nitric oxide level in oxidative hepatic injury. Value = mean ± SD; *n* = 3. *Statistically significant compared to untreated hepatic cells; #statistically significant compared to the control cells (*p* < 0.05, Dunnett’s multiple range *post hoc* test).

Induction of oxidative stress led to a significant (*p* < 0.05) elevation in hepatic nitric oxide (NO) level (Figure 4). Pretreatment of cells with *L. javanica* infusion led to remarkable lower levels of NO in a dose-dependent trend. The infusion presented a better effect on NO level than both ascorbic acid and gallic acid.

**FIGURE 5:**
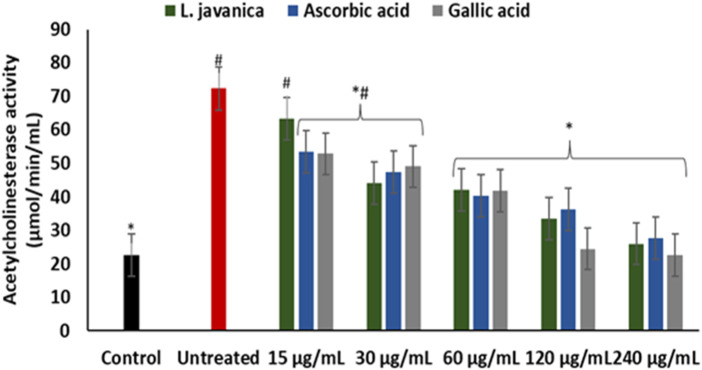
Effect of *L. javanica* on acetylcholinesterase activity in oxidative hepatic injury. Value = mean ± SD; n = 3. *Statistically significant compared to untreated hepatic cells; #statistically significant compared to the control cells (*p* < 0.05, Dunnett’s multiple range *post hoc* test).

There was a significant (*p* < 0.05) elevation in acetylcholinesterase activity on incubation of Chang liver cells with FeSO_4_ (Figure 5). Except at the lowest dose, pretreatment with *L. javanica* infusion led to a significant (*p* < 0.05) dose-dependent suppression of acetylcholinesterase activity and was competitive with those of the standards, ascorbic acid and gallic acid.

**FIGURE 6:**
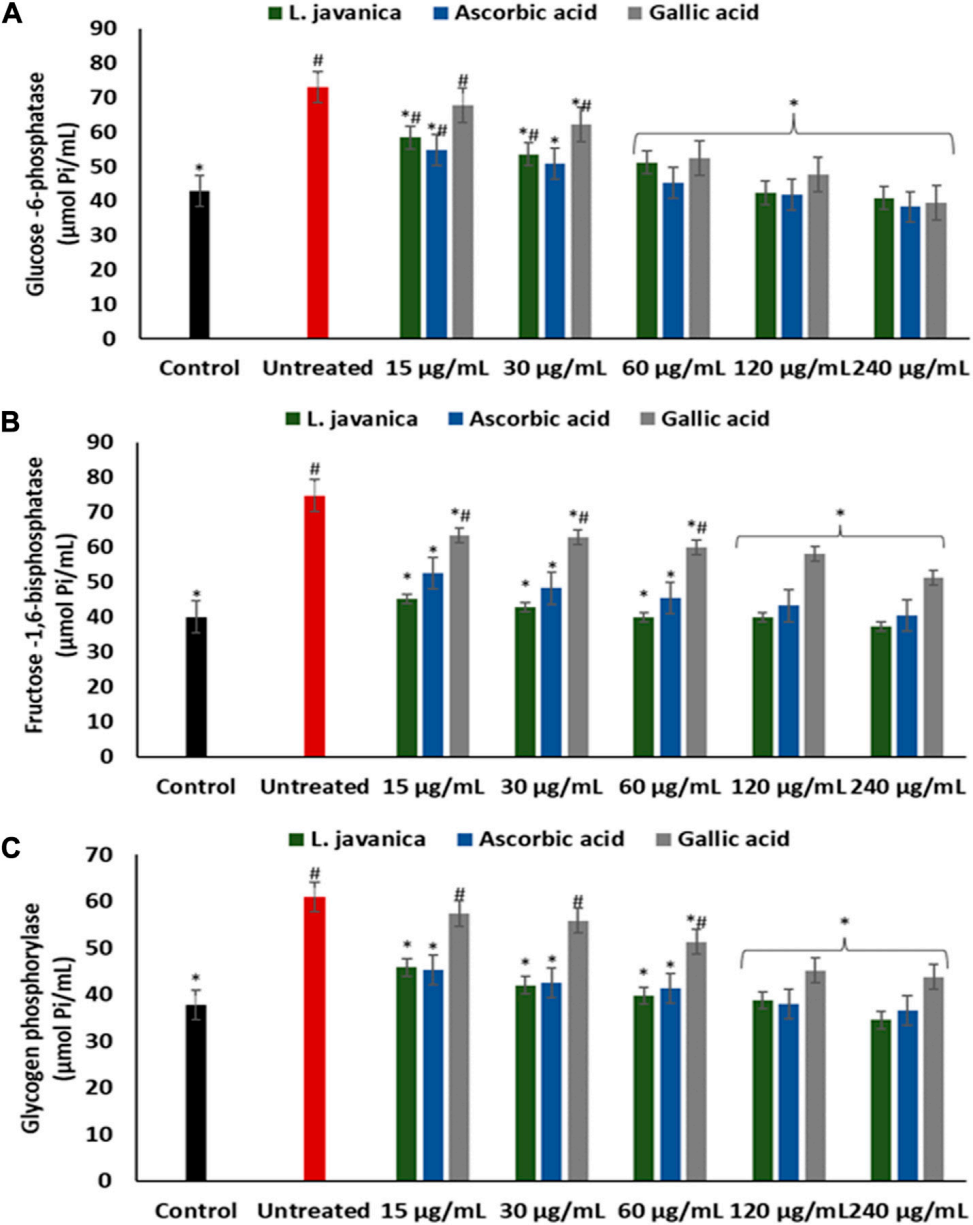
Effect of *L. javanica* on **(A)** Glucose 6 phosphatase, **(B)** Fructose 1,6 bisphosphatase and **(C)** Glycogen phosphorylase activities in oxidative hepatic injury. Value = mean ± SD; *n* = 3. *Statistically significant compared to untreated hepatic cells; #statistically significant compared to the control cells (*p* < 0.05, Dunnett’s multiple range *post hoc* test).

Induction of oxidative injury caused significant (*p* < 0.05) elevation of glucose-6-phosphatase, fructose-1,6-bisphosphatase and glycogen phosphorylase activities as depicted in Figures 6A–C. Pretreatment with *L. javanica* led to significant suppression of the enzyme’s activities in a dose-dependent trend to levels indistinguishable from the normal control.

**FIGURE 7:**
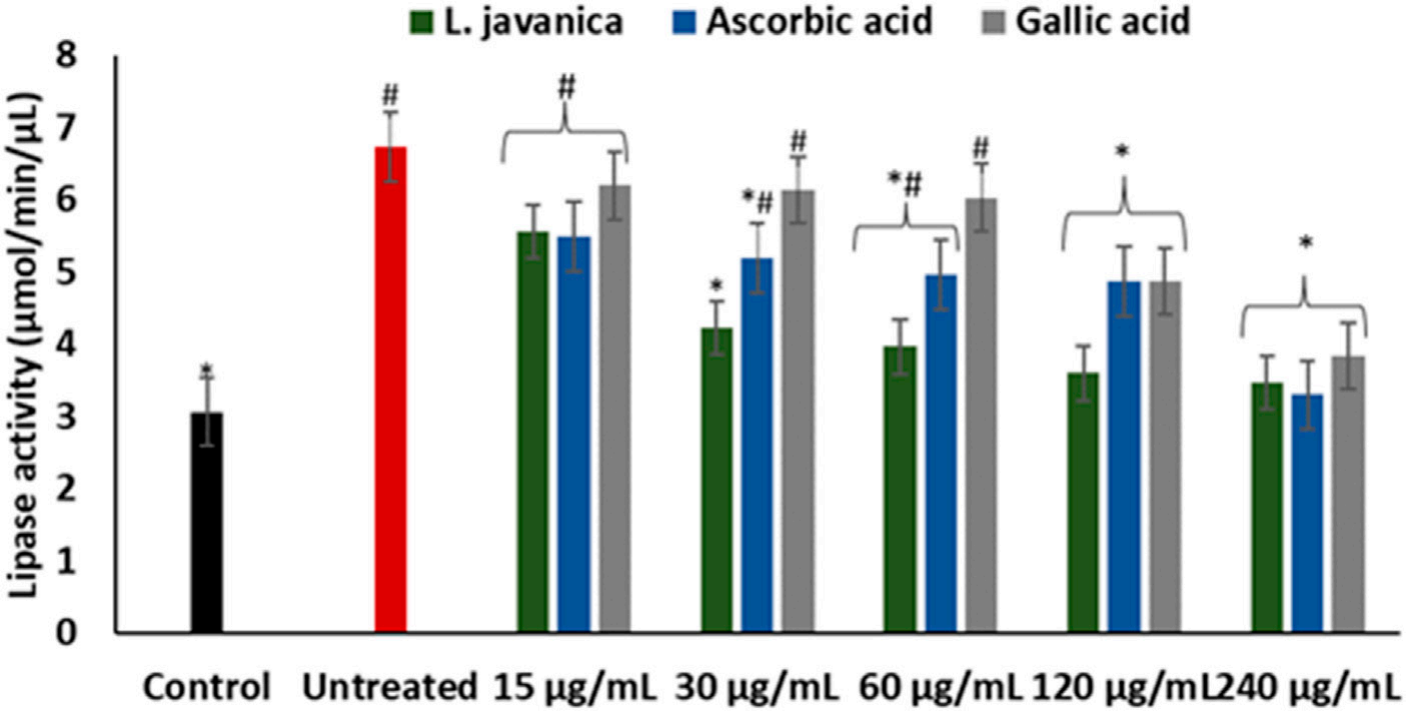
Effect of *L. javanica* on lipase activity in oxidative hepatic injury. Value = mean ± SD; n = 3. *Statistically significant compared to untreated hepatic cells; #statistically significant compared to the control cells (*p* < 0.05, Dunnett’s multiple range *post hoc* test).

As presented in Figure 7, induction of hepatic oxidative injury significantly (*p* < 0.05) elevated the activity of lipase. Incubation of the cells with *L. javanica* significantly (*p* < 0.05) depleted the activity of the enzyme which outperformed the activities of ascorbic acid and gallic acid.

**FIGURE 8:**
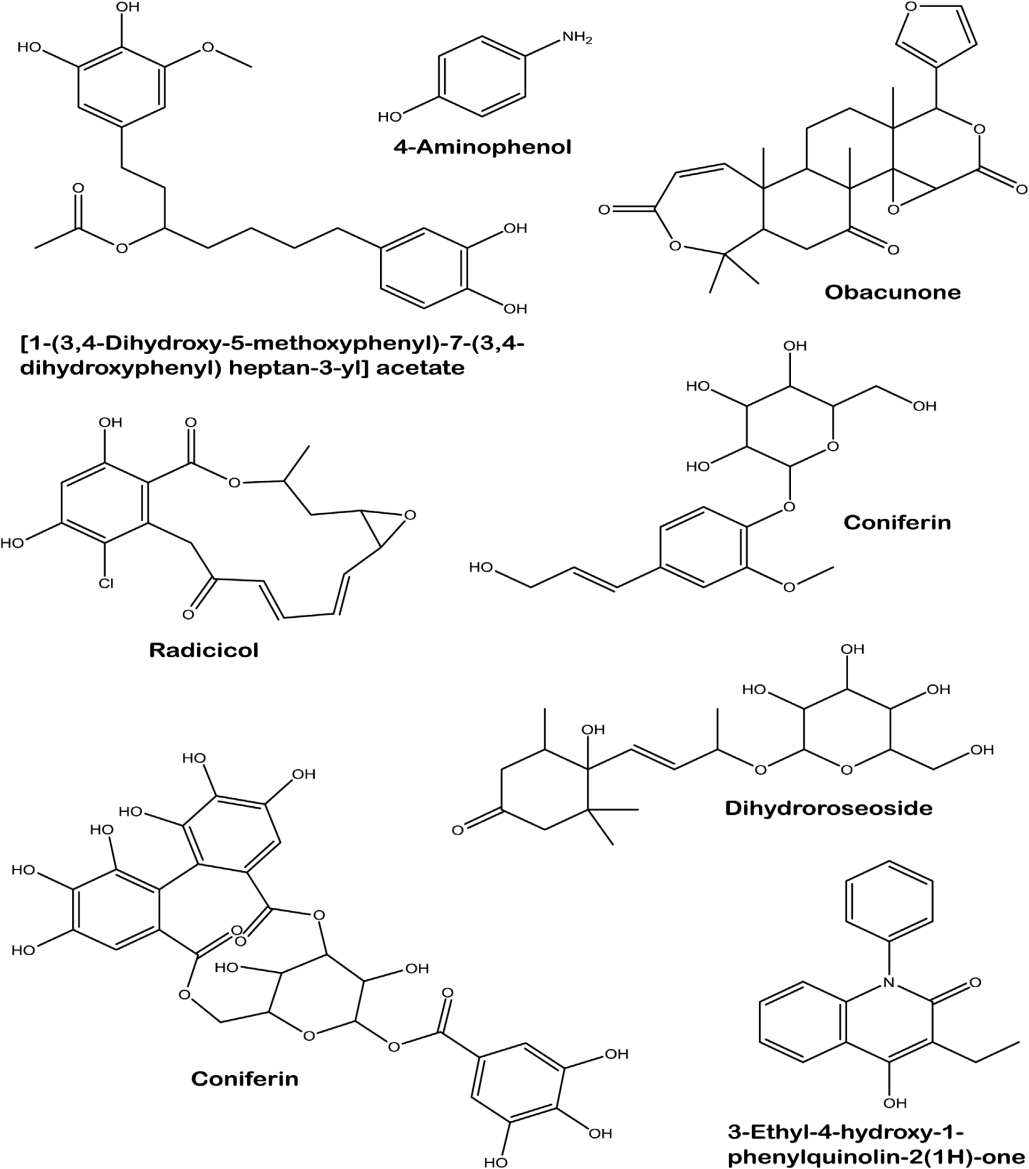
Structures of identified compounds in *L. javanica* infusion.

LC-MS analysis of *L. javanica* infusion revealed the presence of phenolic compound such as amino phenol; tannins such as corilagin; phenolic glycosides such as coniferin and dihydroroseoside and terpenoids such as obacunone and radicol. [1-(3,4-Dihydroxy-5-methoxyphenyl)-7-(3,4-dihydroxyphenyl) heptan-3-yl] acetate and 3-Ethyl-4-hydroxy-1-phenylquinolin-2(1H)-one were also identified in the infusion (Table 2; Figure 8).

**TABLE 2:** Liquid Chromatography Mass Spectrometry (LC-MS) identified chemical compounds of *L*. *javanica*.

Retention time (min.)	Area/Height	Phytoconstituents (suspected)	[M + H] ^+/−^ *m/z*
6.111	15.713	4-Aminophenol	133.10
8.114	20.322	Dihydroroseoside	387.10
10.325	21.875	[1-(3,4-Dihydroxy-5-methoxyphenyl)-7-(3,4-dihydroxyphenyl) heptan-3-yl] acetate	403.05
10.928	13.971	Corilagin	277.10
11.891	17.901	Radicicol	321.10
12.684	19.806	Coniferin	365.10
13.303	14.885	Obacunone	409.20
16.671	16.316	3-Ethyl-4-hydroxy-1-phenylquinolin-2(1H)-one	266.20

**FIGURE 9:**
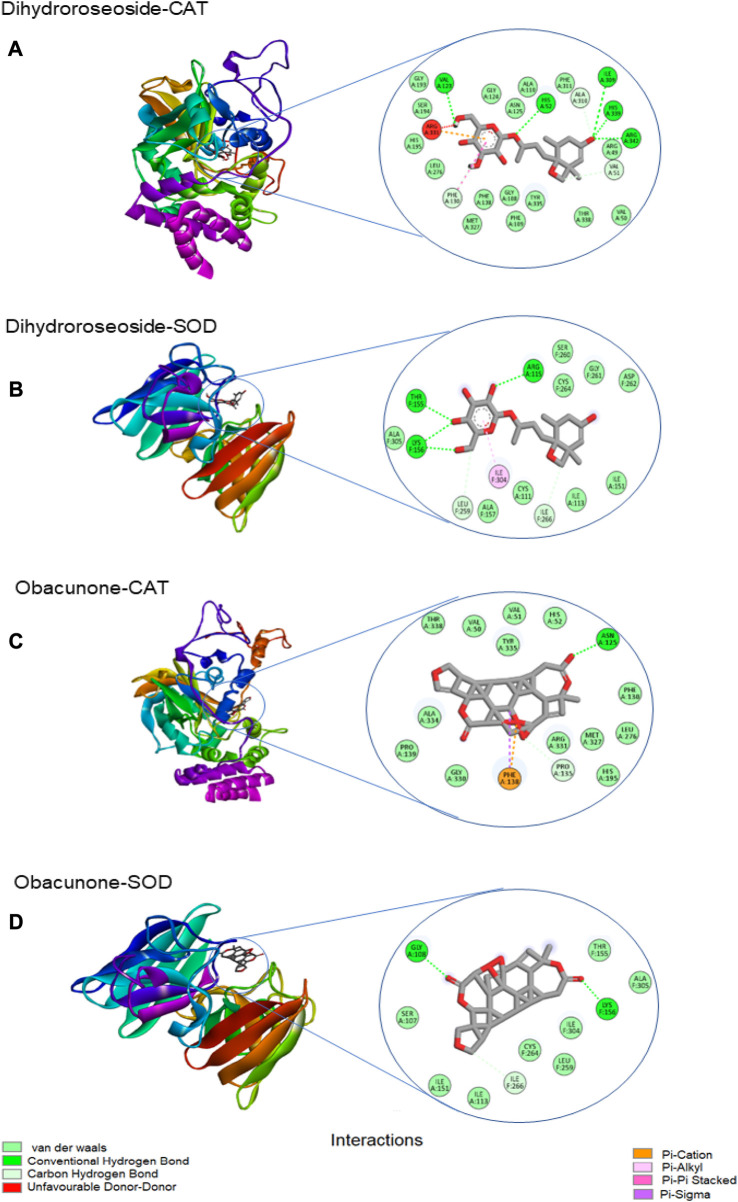
The 3D and 2D images of the molecular interaction of dihydroroseoside with the amino active site of **(A)** catalase and **(B)** SOD; and 3D and 2D images of the molecular interaction of obacunone with active sites of **(C)** catalase, and **(D)** SOD. SOD = superoxide dismutase.

Table 3 shows the free binding energies of the molecular docking of the phytoconstituents of *L. javanica* and antioxidant standards (ascorbic acid and gallic acid) with catalase and SOD, which indicates that dihydroroseoside and obacunone had the highest binding affinity. Figure 8 gives a representation of 3D and 2D images of the molecular interaction of the active site of catalase with dihydroroseoside (Figure 9A), SOD with dihydroroseoside (Figure 9B) and catalase with obacunone (Figure 9C), and SOD with obacunone (Figure 9D). The compounds molecular interacted with the amino acid residues of the binding pocket of the proteins via hydrogen bonds (H-bond), carbon hydrogen bonds, and vanda Waal forces. Dihydroroseoside shared multiple H-bond with both catalase and SOD, while obacunone shared a single and double H-bond with catalase and SOD, respectively.

**TABLE 3:** Binding energies (Kcal/mol) of the phytochemical constituents of *L. javanica* with antioxidant enzymes.

Phytoconstituents	Catalase	Superoxide dismutase
4-Aminophenol	−5.2	−3.7
Dihydroroseoside	−10.5	−7.1
[1-(3,4-Dihydroxy-5-methoxyphenyl)-7-(3,4-dihydroxyphenyl) heptan-3-yl] acetate	−3.6	−2.4
Corilagin	−2.0	−1.7
Radicicol	−5.7	−7.2
Coniferin	−2.0	−1.7
Obacunone	−11.7	−9.8
3-Ethyl-4-hydroxy-1-phenylquinolin-2(1H)-one	−8.7	−6.6
Gallic acid	−7.1	−6.7
Ascorbic acid	−4.8	−5.3

## Discussion

Oxidative hepatic injury is a culprit in the pathological mechanisms that leads to the initiation and development of various liver diseases. Liver diseases have been reported to contribute significantly to socio-economic burden, low quality of life and mortality (Li et al., 2015; Asrani et al., 2019). Medicinal plants constitute a variety of secondary metabolites with inherent antioxidant properties that has been widely reported for the prevention and treatment of hepatic oxidative injury and numerous liver diseases (Stickel and Schuppan, 2007; Chukwuma et al., 2019). In this study, the protective effect of *L. javanica* herbal tea was investigated in iron-induced oxidative hepatic cells injury.

When surplus ROS are generated in the body, they deplete antioxidant levels, leading to a failure to counteract ROS deleterious activities which then result in cellular injury (Pham-Huy et al., 2008). Medicinal plants, including herbal teas are well noted for their rich antioxidant competence, which are ascribed to the presence of prominent phytoconstituents such as phenolics, terpenes, flavonoids, tannins, glycosides, alkaloids and saponins (Mathivha et al., 2020). The antioxidant capacity of these plant constituents are attributed to their ability to act as reducing agents, metal ion chelators, quenchers of singlet oxygen and free radical scavengers (Bendary et al., 2013; Salau et al., 2021). The potent DPPH scavenging activity of *L. javanica* infusion (Figure 1A), coupled with its ability to reduce Fe^3+^ to Fe^2+^ (Figure 1B) indicate the antioxidant pharmacological property of this plant. This can be linked to the high phenolic and flavonoid contents of the plant as well as the presence of LC-MS identified bioactive compounds which include terpenoids, tannins, phenols and glycosides (Figure 8; Table 2). These results corroborates with the previous study of Osunsanmi et al. (2019) who reported that the crude extract of *L. javanica’s* leaves are embedded with varieties of phytochemicals that are a source of natural antioxidants for treating and managing oxidative stress related diseases.

To further buttress the antioxidant and other possible pharmacological potential of *L. javanica* as attributed to its phytoconstituents, its effect on iron induced oxidative hepatic injury were assessed. The liver is an organ that is constantly exposed to oxidative attack due to its critical and numerous physiological roles in the body that also involves generation of ROS as by-products and involvement in ROS-generation reactions (Li et al., 2015; Conde de la Rosa et al., 2022). This includes the liver being the major site of iron storage which makes it a major target for iron toxicity (Pietrangelo, 2016). The ability of iron to exist in dual forms: Fe^2+^ and Fe^3+^, presents it as a major cofactor in many enzymatic redox reactions as well as a potent prooxidant (Erukainure et al., 2017). Thus, hepatic iron overload and/or iron toxicity leads to the generation of reactive oxygen species and reactive nitrogen species, which are responsible for lipid, protein and nucleic acid peroxidation and proinflammation that ultimately contributes the promotion of hepatic oxidative injury and numerous liver diseases (Videla et al., 2003; Milic et al., 2016). The suppressed levels of GSH and NPSH, SOD and catalase activities and increased levels of MDA and NO on induction of hepatic injury (Figures 3A–E; Figure 4) indicate an occurrence of oxidative stress and proinflammation. This corroborates previous study on Fe^2+^ induced oxidative injury in Chang liver cells (Chukwuma et al., 2019). Treatment with the *L. javanica* infusion demonstrated an antioxidant and anti-proinflammatory effect by significantly elevating the levels of GSH and NSPH, SOD and catalase activities and suppressing MDA and NO levels. Thus, indicating a protective effect of *L. javanica* on oxidative hepatic injury. The strong molecular interaction formed between the LC-MS identified phytoconstituents of *L. javanica* and the amino residue active sites of catalase and SOD (Figure 9; Table 3) further indicates the antioxidant potency of the infusion. These properties may be attributed to the synergistic activities of the identified phytochemical constituents.

Cholinergic dysfunction typified by elevated cholinesterase activities has been reported as one of the instigators of hepatotoxicity as it incites hepatic cells inflammation (Erukainure et al., 2021b), Altered acetylcholinesterase level has been reported as a useful biomarker for liver disease (García-Ayllón et al., 2006). Studies have linked oxidative stress and proinflammation to a rise in acetylcholinesterase activities (Bondok et al., 2013; Rodríguez-Fuentes et al., 2015). In the present study, the elevated activity of hepatic acetylcholinesterase (Figure 5) on induction of hepatic injury conforms with oxidative stress occurrence (Figure 3) and increased NO level (Figure 4). Cholinesterase inhibitors are targets for protecting against and treating liver diseases (Steinebrunner et al., 2014). The depleted activity of acetylcholinesterase on treatment with *L. javanica* infusion insinuates a protective effect of the herbal tea against cholinergic dysfunction in oxidative hepatic injury.

The liver plays a pivotal function in the maintenance of glucose homeostasis and metabolism. Perturbations in hepatic glucose level is implicated in development of several liver diseases (Ding et al., 2018; Zhang et al., 2019). Oxidative stress contributes to disturbances in carbohydrate metabolism, which can lead to perturbed energy metabolism (Yazdi et al., 2019). Increased activities of enzymes involved in hepatic gluconeogenesis and glycogenolysis leads to the generation of excess glucose. Glucose toxicity characterised by hepatic glucose accumulation promotes hepatic oxidative stress which results in severe liver oxidative injury and eventual hepatic cell death (Chandrasekaran et al., 2012; Mota et al., 2016). Reports have shown the protective property of various plant-based antioxidants in scavenging free radical and the improvement of liver carbohydrate metabolism (Yazdi et al., 2019; Erukainure et al., 2021a; Olofinsan et al., 2022). This is in accordance with the ability of *L. javanica* herbal infusion to significantly reduce the activities of glucose-6-phosphatase, fructose-1,6-bisphosphatase and glycogen phosphorylase in oxidative hepatic injury (Figures 6A–C), suggesting a hepatoprotective and hepatic metabolic function-improving potential of the herbal tea infusion.

The damaging effect of oxidative stress in the development of liver diseases cannot be overemphasized. ROS can induce lipid peroxidation by attacking the polyunsaturated fatty acids of lipid membrane and impair cellular functions (Barrera, 2012). Increased level of lipase activity in the liver has been reported in hepatotoxicity due to its catalyzed excessive breakdown of triglycerides, which causes accumulation of hepatic free fatty acids (Erukainure et al., 2021a). Thus, oxidative attack of vulnerable fatty acids progresses oxidative injury. Cytotoxic products generated from hepatic lipid peroxidation such as malondialdehyde (MDA) have been implicated in hepatic fibrogenesis (Koruk et al., 2004). The elevation of lipase activity on induction of oxidative injury (Figure 7) with concomitant increased MDA level (Figure 3D) may indicate progressive oxidative hepatic injury and development of liver disease. The ability of *L. javanica* infusion to significantly suppress hepatic lipase activity may further suggest the protective effect of the herbal infusion against oxidative hepatic injury.

Phytopharmaceuticals play a key role in medical practice for various diseases treatment and management. However, in addition to their therapeutic properties, many medicinal herbs may contain potential toxic properties that may cause more harm to human health (Lombardi et al., 2017). Cytotoxicity studies is thus a crucial step in determining the safety and therapeutic properties of plants and plant-derived compounds for oral pharmacological agent development (Beseni et al., 2022). The cell viability effect of *L. javanica* infusion (Figure 2) portrays a non-cytotoxic effect on liver cells. Thus, implying the safety of the *L. javanica* herbal tea on hepatic cells.

## Conclusion

Results from this study demonstrated that *L. javanica* conferred hepatoprotection against oxidative hepatic injury by mitigating oxidative stress and cholinergic dysfunction as well as improved impaired glucogenic and lipase enzymes activities. These biological activities may be attributed to the synergistic effect of the identified phytoconstituents. Thus, these results may support the ingestion of *L. javanica* herbal tea as potential curative agent for liver diseases. Further *in vivo* and molecular studies are required to unravel the mechanisms by which *L. javanica* brings about its hepatoprotective effect in hepatic oxdative injury. The limitation of the present study is the non-use of commercial standard for the LC-MS analysis. We therefore propose the use commercially available standards for future characterization of *L. javanica* phytoconstituents.

## Data Availability

The original contributions presented in the study are included in the article/Supplementary material, further inquiries can be directed to the corresponding author.
